# Factors facilitating and inhibiting the social participation of the elderly in health-oriented activities in Shiraz, Southern Iran

**DOI:** 10.1186/s12877-023-03892-4

**Published:** 2023-03-27

**Authors:** Marzie Tajik Jalali, Yaser Sarikhani, Fatemeh Askarian, Milad Ahmadi Marzaleh, Seyede Maryam Najibi, Sajad Delavari

**Affiliations:** 1 Health Human Resources Research Center, School of Health Management and Information Sciences, Shiraz University of Medical Sciences, Shiraz, Iran; 2 Research Center for Social Determinants of Health, Jahrom University of Medical Sciences, Jahrom, Iran; 3 Department of Health in Disasters and Emergencies, Health Human Resources Research Center, School of Health Management and Information sciences, Shiraz University of Medical Sciences, Shiraz, Iran

**Keywords:** Elderly, Social Participation, Health-oriented activities, Iran

## Abstract

**Background:**

The social participation (SP) of the elderly is one of the factors that contribute to the improvement of their well-being. SP, one of the most important factors of active ageing, is mainly influenced by a number of facilitating or inhibiting factors.

**Aims:**

This study aimed to identify the factors that prevent and facilitate the SP of the elderly population in Iran.

**Methods:**

A cross-sectional study carried out in Shiraz, southern Iran in 2021. Participants were selected using a convenience sampling method. Shiraz is divided into 11 districts and the largest park in each district is selected for data gathering. The questionnaires were completed by 612 people aged over 60. Data were collected using the Canadian Elderly Survey Project scale and a health-related lifestyle questionnaire and were analyzed using t-test, ANOVA, Pearson’s correlation, and ANCOVA.

**Results:**

The mean SP score of the elderly in Shiraz was 24.2 out of 60, which is below the midpoint. The results of the covariance analysis revealed that the SP had a significant relationship with the experience of physician consultation, cost barriers, age, marital status, income level, and education level (P < 0.001). Moreover, the results of Pearson correlation revealed a significant correlation between SP and different dimensions of health-oriented activities (< 0.001).

**Conclusions:**

This study revealed that the main barriers to older people’s participation in health-related activities are cost and access barriers, such as transportation issues. Moreover, higher income level and higher educational attainment have been recognized as the main facilitators of SP in the elderly. In this regard, it can be suggested to apply a combination of health promotion strategies, financial support programs, and development of optimal transportation infrastructure to increase the SP of the elderly.

## Introduction

Since the beginning of the new millennium, the increase in the elderly population has become one of the greatest challenges facing all countries in the world [1]. Old age refers to a period of human life when a person’s physical strength becomes weak and incapacitated due to changes in the state of the body cells. Based on chronological age, old age is generally assumed to be 60 or 65 years and beyond [2, 3]. It is predicted that by 2060, for the first time in human history, the number of older people will outnumber children under 15 [4]. Statistical and demographic indicators show that the aging phenomenon in Iran has started and is expanding [5].

Over the past decade, due to the growth of the elderly population and the impact of their health status on various social and economic aspects, investigating the factors leading to improvements in the health and well-being of older people has become the top priority of health systems in most countries around the world [6]. In old age, people’s psychophysical state are more influenced by factors such as physical activity levels, social interactions, and their attitudes toward old age [7]. To address the negative effects of population ageing, it is therefore necessary to identify the factors that determine the health and well-being of the elderly. In this regard, the social participation (SP) of the elderly is one of the factors that contribute to the improvement of their well-being [8]. Because SP is an organized process in which people are defined by their specific, collective, conscious, and voluntary actions, it ultimately leads to self-actualization of the individual [9]. The importance of SP increases when this participation takes place in the context of health-oriented activities, which is considered as one of the most important criteria for determining peoples’ health status [10]. The study by Ma et al. shows that SP in health-oriented activities prevents functional disabilities, mental disorders and cognitive impairments, and significantly delays premature mortality in the elderly [11]. Furthermore, some studies have shown that SP can have positive effects such as reducing the risk of depression, improving mental health, and improving the overall health of the elderly [12, 13].

Since one of the most important aspects of SP is its positive impact on mental well-being, it is extremely essential to make efforts to increase SP in the aging population [14]. SP of the elderly, one of the most important drivers of active ageing, is mainly influenced by a number of underlying factors which are considered as facilitators and inhibitors. Facilitating factors are potential factors that can lead to an increase in SP, and inhibiting factors are various factors that can potentially lead to a decrease in SP in the elderly. Facilitating and inhibiting factors are classified into several categories, including economic factors, access factors, and personal characteristics [8]. In this regard, several studies have investigated the relationship between demographic factors and the social participation of the elderly [15–17]. Moreover, in a systematic review, Townsend et al. identified four major issues that inhibit and facilitate older people’s social participation, including demographic factors (such as age and socioeconomic status), individual/internal factors (such as motivation and health), environmental infrastructure (such as access, transport, and neighborhood), and social networks (especially the size of the existing network) [18]. However, the relationship between these factors and health-oriented activities is an important issue addressed in this study. Since the identification of these factors is of great importance for improving the health and well-being of the elderly [19], Since this study aimed to identify these factors in Shiraz, southern Iran. The findings could help to develop long-term plans to address the negative effects of aging in the Iranian population.

## Methods

This descriptive-analytical study was conducted in 2021. The target population in this cross-sectional study was the people over the age of 60 living in the 11 municipal districts of Shiraz city, the capital of Fars province in southern Iran.

Based on estimates from a similar study in which the mean score for SP of the elderly was 6.71 ± 4.01 [8], and taking into account type 1 error of 5%, precision of 0.48, and a design effect of 2, the sample size was calculated to be 530. Finally, considering a 15% of nonresponse, the final sample size was estimated at 610. We applied a convenience sampling method to select the participants. In each district of the city, the largest park as the most common meeting place for the elderly was chosen for data collection. Accordingly, eleven large city parks were selected. On this basis, 60 participants from each park were considered in proportion to the number of the estimated sample size (660 questionnaires in total). With multiple referrals, we managed to complete 612 questionnaires. The rate of non-response was 7.27%, mainly due to the cognitive difficulties of the elderly in completing the questionnaires. Living in the city of Shiraz, being over 60, Iranian citizenship, ability to communicate, and completing an informed consent form were considered inclusion criteria. Four interviewers who had previously received the necessary training were employed to gather the data. Each interviewer was selected to complete the questionnaires in two or three parks.

Data collection tools included two questionnaires on SP and health-oriented activities. In this study, we used the SP questionnaire of the Canadian Elderly Survey Project. The Persian version of this questionnaire has been validated in a study by Darvishipour et al. [20]. This questionnaire contains 21 items, seven of which address personal characteristics such as gender, age, marital status, education level, income, health information, and ability to use social networks. There are nine questions on group work, participation in religious ceremonies, group sports, educational and cultural activities, activities in social and charitable centers, and group entertainment. These questions had a five-point Likert scale design and responses included zero (never), one (at least once a year), two (at least once a month), three (at least once a week), and four (at least once a day). The total score of the items in this part is considered the SP score of the elderly. In addition, the questionnaire contains five yes-no type questions on the barriers to the SP of elderly. Based on these questions, the barriers are divided into five categories including, cost barriers (cost of participation in social activities, income and financial status), access-related barriers (transportation, unavailability of areas of activity, and physical inaccessibility due to geographic location), health-related barriers (fear or concern for safety, illnesses, and health problems), non-health personal barriers (reluctance to go out alone, inappropriate time of activity, communication difficulties, family or personal commitments, lack of planning, lots of daily activities, and boredom), and other barriers (obstacles other than the above four items).

For the health-oriented activities was used a health-related lifestyle questionnaire which was validated in the study of Sharafi et al. [8]. This questionnaire contains 20 questions, including four items about weight control and diet, one item on prevention, four items about physical well-being, three items on exercise, four items on mental well-being, and four items about social well-being. The questions were scored on a Likert scale from 0 (very little) to 5 (very much).

The questionnaires were completed in person by one of the researchers using a tablet. To this end, the questions were explained to the elderly and the answers were recorded digitally in questionnaires. Completing the questionnaires for each participant took an average of 20 min. Incomplete questionnaires were excluded from the study.

### Statistical analysis

We used frequency, mean ± standard deviation to describe the data. Independent sample t-test and ANOVA were applied to compare the mean score of SP of elderly in different groups. Moreover, covariance analysis was applied to adjust the effect of study variables. The variables with a significance level of more than 0.20 in the univariate analysis were not entered into the covariance analysis. Moreover, we used a Pearson correlation test to investigate the association between SP and different dimensions of health-oriented activities. The significance level of the statistical tests was set at 0.05. We applied SPSS version 19.0 (SPSS Inc., Chicago, IL) to analyze the data.

## Results

Of the 660 questionnaires distributed, 612 were fully completed (response rate 92.7%). Most of the participants were male (66.8%), married (57.7%) and had a chronic disease (57%). The average age of the participants was 69.11 ± 4.9. Table 1 represents the demographic characteristics of the participants.

**Table 1:** Demographic characteristics of the participants

Variable		Frequency (%)
Gender	Male	409(66.8)
	Female	203(33.2)
Marital status	Single	52(8.5)
	Divorce	55(9.0)
	Widowed	152(24.8)
	Married	353(57.7)
Education level	Illiterate	85(13.9)
	Read and write (Basic)	237(38.7)
	Under high school	126(20.6)
	High school	85(13.9)
	Collegiate	79(12.9)
Income level	Very low	17(2.8)
	Low	258(42.2)
	Medium	205(33.5)
	High	48(7.8)
	Very high	84(13.7)
Chronic disease	Yes	349(57.0)
	No	263(43.0)
Total	-	612(100)

The results of Pearson correlation analysis indicated that there was a significant correlation between the mean score of SP and different dimensions of health-oriented activities (P < 0.001). The findings of the correlation analysis are provided in Table 2.

**Table 2:** The association between the mean score of SP of elderly and different dimensions of health-oriented activities scale

Dimensions	Mean ± SD	Pearson Correlation	P-value
Weight control and diet	8.80 ± 3.55	0.89	< 0.001
prevention	2.94 ± 0.64	0.77	< 0.001
Physical wellbeing	11.13 ± 3.93	0.63	< 0.001
Exercise	7.20 ± 2.86	0.88	< 0.001
Mental wellbeing	11.08 ± 2.51	0.92	< 0.001
Social wellbeing	10.84 ± 3.02	0.75	< 0.001

The results showed that the mean score of SP of the elderly in Shiraz is 24.2 out of 60, which is below the midpoint. As shown in Table 3, the results of t-test indicated that there were no significant differences between the mean score of SP based on the status of having health-related barriers, personal barriers, and other types of barriers to SP. However, the results of t-test indicated that there was a significant relationship between the score of SP and gender, having chronic diseases, history of hospitalization in the past six months, having access-related barriers and cost barriers, and having the history of doctor’s consultation within the past seven days (P < 0.001). Nevertheless, after adjustment by analysis of covariance, the relationship remained significant only for cost barriers and having the history of doctor’s consultation within the past seven days (P < 0.001).

Similar to the results of the covariance analysis, the results of ANOVA revealed that the mean score of SP had a significant relationship with the age, marital status, income level, and education level (P < 0.001). The results of the post-hoc comparison with the Scheffe’s test indicated that the mean score of SP decreased significantly with age (P < 0.001). Moreover, the post-hoc test showed a direct relationship between the mean score of SP and the increase in education level (P < 0.001). The results of the Scheffe’s test showed that as the income level increased, the mean score of SP increased significantly (P < 0.001). Finally, the results of the post-hoc analysis showed that the single elderly had a higher score of SP compared to the other marital status groups (P < 0.001). Table 3 shows the results of univariate and covariance analyses.

**Table 3:** Unadjusted and adjusted relationship between SP of elderly and the study variables

Variables		N	Mean ± SD	Unadjusted P-value	Adjusted P-value
Gender	Male	409	23.20 ± 9.32	< 0.001	0.436
	Female	203	26.43 ± 9.40		
Chronic Disease	Yes	349	21.97 ± 7.08	< 0.001	0.522
	No	263	27.33 ± 11.22		
Hospital admission	Yes	195	21.96 ± 5.43	< 0.001	0.098
	No	417	25.35 ± 10.69		
Access-related barriers	Yes	124	29.54 ± 10.35	< 0.001	0.054
	No	488	22.94 ± 8.75		
Health-related barriers	Yes	270	24.61 ± 9.48	0.723	---
	No	342	24.01 ± 9.46		
Personal barriers	Yes	366	24.58 ± 9.71	0.327	---
	No	246	23.82 ± 9.09		
Other barriers	Yes	203	24.00 ± 9.64	0.535	---
	No	409	24.41 ± 9.39		
Cost barriers	Yes	391	20.28 ± 6.26	< 0.001	< 0.001
	No	221	31.33 ± 10.06		
Physician consultation	Yes	232	30.33 ± 9.31	< 0.001	< 0.001
	No	380	20.58 ± 7.54		
Age	< 69	452	25.42 ± 9.81	< 0.001	< 0.001
	70–79	96	25.21 ± 7.08		
	> 80	64	14.75 ± 0.97		
Marital status	Single	52	36.76 ± 5.49	< 0.001	< 0.001
	Divorce	55	24.47 ± 9.87		
	Widowed	152	19.49 ± 6.29		
	Married	353	24.46 ± 9.29		
Income level	Very low	17	18.00 ± 1.65	< 0.001	< 0.001
	Low	258	17.72 ± 1.84		
	Medium	205	25.88 ± 8.25		
	High	48	25.43 ± 8.73		
	Very high	84	41.08 ± 1.97		
Education level	Illiterate	85	16.43 ± 2.54	< 0.001	< 0.001
	Basic	237	18.00 ± 2.16		
	Under HS	126	26.03 ± 8.25		
	High school	85	31.44 ± 5.81		
	Collage	79	41.02 ± 2.02		

## Discussion

Given the growing proportion of the elderly in most countries of the world, increasing the SP of the elderly will improve the general health status of society [6]. Accordingly, the present study was conducted with the aim of determining the facilitators and inhibitors of SP in the elderly of Shiraz.

The results of the present study showed that there is a positive association between SP and different dimensions of health-oriented activity among elderly. Several studies, consistent with this research, have shown that SP of older adults has positive effects on various health outcomes, including mortality, mental health, functional and cognitive skills, and quality of life [11, 21–24]. Indeed, SP leads to improvements in happiness [25, 26], health behavior [27], quality of life [28, 29], and satisfaction in older adults [30, 31]. Therefore, it should be the concern of policymakers in the field of aging to pay attention to the importance of active SP to maintain and improve the health of older people.

In this study, the mean score of SP of elderly was 24.2 out of 60, which was below the midpoint of SP and shows that a significant number of the elderly do not participate in social activities due to various barriers. Consistent with the results of our study, similar studies conducted in Tehran [20] and Kerman [32] reported that the mean score of SP in the elderly was 15.96 and 6.71, respectively, which were lower than average level. This is despite the fact that SP is one of the important and influential components of social and economic development in a dynamic society. Moreover, participating in many affairs of society during the old age period contributes to successful ageing and in turn results in the development of elderly and society [33]. These facts underline the need for public infrastructures that facilitate the SP of the elderly, which requires special attention as a prerequisite in the national health system policies.

According to the results of this study, the most common barrier to SP of older adults was the financial inability to pay expenses. In similar study, Feng et al. (2020) reported that high-income groups were almost twice as likely to participate in social activities as low-income groups [34]. Based on this research findings, Sun et al. (2021) also confirms the relationship between higher income and participation in more social activities among older adults [35]. This result was also confirmed in the study by Sharafi et al. (2022), who found that the economic problems and the insufficient social security of the elderly in Iran are among the main challenges that reduce their SP [8]. This point underlines the importance of reviewing social security policies and strengthening the government’s economic structure in the area of ageing. Zhang et al. (2022) suggests that higher income levels are often associated with better nutrition, housing, medical care, and increased health awareness, leading to better health outcomes. A higher SP of elderly with higher incomes can therefore be influenced by the mutual influence of these factors [12]. In general, wealthier elderly or those who are from more developed societies have more opportunities and incentives to participate in social and political activities [8]. It should also be noted that a higher income level removes cost issues which are among the major barriers of SP.

The second major barrier that negatively impacted older adults’ SP was the access barrier. This barrier includes transportation issues, unavailability of activity areas, and physical inaccessibility due to geographic location. In this regard, Levasseur et al. (2010) proposed to remove access barriers as one of the effective measures to increase the SP of the elderly [36]. Amiri et al. (2017) also states that the availability of suitable and safe transport services for the elderly along with safe environmental facilities, such as the presence of suitable sidewalks and roads, the presence of ramps and support structures, play a crucial role in SP [37]. Therefore, policy makers in different fields, especially urban planners, should be given special attention regarding these issues.

The results of this study, consistent with the findings of some other studies [20, 32], showed that the mean score of SP in the elderly decreases significantly with age. It seems that the inverse relationship between age and SP may be due to the fact that pain and psychophysical complications increase in the elderly with the age. These physical-functional limitations and the lack of functional independence in daily activities can influence the withdrawal of the elderly from the society and the reduction of their SP. However, a study by Willie-Tyndale et al. (2016) among the elderly in Jamaica reported no association between SP and age [38]. The different findings of the above study may be due to the measurement of SP in organized social group meetings, such that older adults are often unable to participate in organized activities.

The results of the present study showed that highly educated older adults have a better SP than less educated elderly. This means that education can be considered as a factor that promotes the SP of the elderly. In this regard, Katagiri & Kim(2012) points out in her study that SP was higher in older women who had completed their high school years than in women with less than high school education [14]. It seems that as the level of education among the elderly increases, self-confidence and communication skills will increase among them. It also changes the way they think about relationships and SP in a positive way [11]. Ultimately, these changes may lead to an increased desire for SP in more educated older adults. In contrast, the study by Marsh et al. (2018) in rural Sri Lanka reported a weak relationship between education level and SP of the elderly when controlling for other factors [16]. Limitations of access to social activities due to geographical location may justify the contradictory result of this study.

The results of this study showed that the SP score was higher in elderly people living alone. Contrary to the current study, Darvishpour et al. (2014) argues that married older people have higher SP than unmarried people due to their better mentality [20]. On the other hand, in agreement with the results of the current study, Sharafi et al. (2022) concluded that SP is higher in single elderly. The reason for this difference can be attributed to fewer family problems and responsibilities of the elderly living alone [8]. However, the existing differences may be caused by the contextual differences of the communities, which remains as an area for further research.

### Limitations of the study

Data collection in the present study took place during the outbreak of the COVID-19 pandemic, which not only made access to participants more difficult, but also lengthened the data collection process. On the other hand, concern about the risk of contracting COVID-19 may have influenced participants’ experiences and statements. Furthermore, since the study population was made up of elderly people who were difficult to reach, we necessarily used convenience sampling. Therefore, due to the lack of a sampling framework, it was not possible to take advantage of random sampling. Moreover, a greater proportion of older adults in the study were younger than 69, which may be because access to older adults required home visits. Therefore, the SP score may not be estimated accurately. Finally, we explored the quantity of SP, while the quality of SP of elderly is also important and should be investigated in future studies.

## Conclusion

The results of this study showed that the main obstacles to older adults’ participation in health-related activities are cost and access barriers, such as transportation issues. Since the removal of these obstacles can play a useful role in increasing the PS of the elderly, it is recommended to financially support the elderly and to adopt appropriate strategies to create sufficient and optimal transport infrastructures. The results also showed that the health status of the elderly is one of the factors affecting their SP. Consequently, it is necessary to promote the health of the elderly through supportive measures.

## Data Availability

The data that support the findings of this study are available on request from the corresponding author.
